# Effects of Valproate Monotherapy on the Oxidant-Antioxidant Status in Mexican Epileptic Children: A Longitudinal Study

**DOI:** 10.1155/2018/7954371

**Published:** 2018-12-04

**Authors:** Eduardo Beltrán-Sarmiento, Cindy K. Arregoitia-Sarabia, Esaú Floriano-Sánchez, Roberto Sandoval-Pacheco, Diana E. Galván-Hernández, Elvia Coballase-Urrutia, Liliana Carmona-Aparicio, Eduardo Ramos-Reyna, Juan Rodríguez-Silverio, Noemí Cárdenas-Rodríguez

**Affiliations:** ^1^National Institute of Pediatrics, Laboratory of Neurosciences, 04530, Mexico; ^2^National Polytechnic Institute, Section of Research and Graduate Studies, Mexico 11340, Mexico; ^3^University of the Army and Air Force, Secretary of National Defense, Military Graduate School of Health, 11200, Mexico

## Abstract

Epilepsy is a neurological disorder that can produce brain injury and neuronal death. Several factors such as oxidative stress have been implicated in epileptogenesis. Valproic acid (VPA) is a widely used drug for the treatment of epilepsy, but the mechanisms underlying these benefits are complex and still not fully understood. The objective of this study was to evaluate, for the first time, the effects of VPA on the oxidant-antioxidant status in Mexican epileptic children before and after 6 or 12 months of treatment with VPA by determining the activities of several plasmatic antioxidant enzymes (glutathione reductase (GR), glutathione peroxidase (GPx), superoxide dismutase (SOD), and catalase (CAT)) and oxidant marker (malondialdehyde (MDA), hydrogen peroxide (H_2_O_2_), 8-hydroxy-2-deoxyguanosine (8-OHdG), and 3-nitrotyrosine (3-NT) levels) profiles. The possible relationships between these markers and some clinicopathological factors were also evaluated. Plasma samples were obtained from the peripheral blood of 16 healthy children and 32 patients diagnosed with epilepsy, and antioxidant/oxidant markers were measured spectrometrically. Significant decreases in all antioxidant enzyme activities, with the exception of GPx, and increases in all oxidant markers in epileptic subjects versus healthy children were observed. Interestingly, all these effects reverted after VPA monotherapy, although the results were different depending on the treatment period (6 or 12 months). These changes were contingent upon brain imaging findings, type of epilepsy, etiology of epilepsy, and the efficacy of 6 months of VPA monotherapy. Significant and positive correlations of GPx and SOD activities and H_2_O_2_ and 8-OHdG levels with the age of children at the beginning of treatment were observed. H_2_O_2_ levels were also positively correlated with number of seizures before VPA monotherapy. VPA showed significant antioxidant effects decreasing seizure activity, possibly depending on the presence of cerebral structural alterations, treatment time, and age.

## 1. Introduction

Epilepsy is a global disease and the most common chronic neurologic disorder. Approximately 70 million people have epilepsy worldwide, and approximately 90% of them are in developing regions [1, 2]. Epilepsy is identified by the International League Against Epilepsy by the following features: (1) at least two unprovoked (or reflex) seizures occurring more than 24 h apart, (2) one unprovoked (or reflex) seizure and a probability of additional seizures similar to the general recurrence risk (60% or higher) after two unprovoked seizures occurring over the next 10 years, and (3) a diagnosis of an epilepsy syndrome [3].

In Mexico, approximately 1.5 to 2 million inhabitants suffer from epileptic crises, with an estimated incidence of just over 100 patients per 1,000,000 inhabitants [4, 5]. Central nervous system infections, family history, trauma, neuronal diseases, and malformations are the most common causes of epilepsy. However, some studies conducted in Mexico and Guatemala have demonstrated neurocysticercosis and traumatic brain injury in 30% of patients with epilepsy in rural areas [6]. Experimental and clinical studies show that epilepsy is characterized by many pathogenic mechanisms primarily related to functional alterations in neuronal and neurovascular units and by an imbalance between excitatory (glutamate) and inhibitory (*γ*-aminobutyric acid) neurotransmission [7, 8]. Recently, using microarray technology, our group showed that the immune response, inflammation, and oxidative stress (OS) pathways are mainly activated in Mexican epileptic children [9]. Antiepileptic drugs (AEDs) are used to treat epilepsy, and they mainly target ion channels and inhibit neuronal excitability. Valproic acid (VPA) is a common first-line AED to control seizures in the pediatric population [10].

VPA is a branched short-chain fatty acid that is also used for treating migraines and bipolar disorder [11]. In epilepsy control, VPA acts via many mechanisms, the most predominant being the enhancement of the inhibitory GABAergic activity and the inhibition of glutamatergic transmission modulating sodium and potassium channels [12, 13]. Several studies have demonstrated that VPA can exert a neuroprotective effect in experimental models and humans by increasing antioxidant defense and decreasing oxidant metabolite production [14–24]. Based on the above data, the objective of this study was to evaluate, for the first time, the effects of VPA on the oxidant-antioxidant status in Mexican epileptic children before and after 6 and 12 months of treatment with this AED by determining plasmatic antioxidant activities and oxidant marker profiles (glutathione reductase (GR), glutathione peroxidase (GPx), superoxide dismutase (SOD), and catalase (CAT) activities and malondialdehyde (MDA), hydrogen peroxide (H_2_O_2_), 8-hydroxy-2-deoxyguanosine (8-OHdG), and 3-nitrotyrosine (3-NT) levels). Moreover, we evaluated the possible relationships between antioxidant or oxidant markers and some clinicopathological factors.

## 2. Material and Methods

### 2.1. Biological Samples

We followed the methods of patient recruitment and sample obtaining and processing described in [9]. The samples were collected from the Service of Emergency, Military Hospital of Specialties of the Woman and Neonatology and of the Electroencephalography Laboratory of Central Military Hospital, Secretary of National Defense (“Secretaría de la Defensa Nacional, SEDENA”) in Mexico City, Mexico, from December 2015 to April 2018. Samples of peripheral blood in EDTA were obtained from 16 healthy children and 32 patients diagnosed with epilepsy and used for plasma isolation. Ethical approval was provided by the Bioethics in Research Committee of the Military Hospital of Specialties of the Woman and Neonatology (registration number 35); the Research and Bioethics Committees of the Hospital Central Military, SEDENA (registration number C.INV.-131); and the Research and Ethics Committees of the National Institute of Pediatrics (registration number 034/2013).

Data on clinical characteristics and anthropometric measures in patients were used in accordance with medical protocols of the Pediatric Service of the Military Hospitals. The human experimentation guidelines of these committees were followed, and written informed consent was obtained from each participant. The study groups were as follows: (a) healthy children (controls), (b) epileptic children before VPA monotherapy (drug-free stage), (c) epileptic children after 6 months of VPA monotherapy, and (d) epileptic children after 12 months of VPA monotherapy.

The inclusion criteria were peripheral blood samples from newly admitted pediatric patients who attended the Military Hospital of Specialties of the Woman and Neonatology and Central Military Hospital, SEDENA, from 2016 to 2018; pediatric patients diagnosed with epilepsy or some epileptic syndrome; untreated pediatric patients for the first sampling; the use of VPA as the only AED (monotherapy); and 12 months of monotherapy without interruption. The exclusion criteria were as follows: pediatric patients with chronic diseases such as hematological, cardiac, hepatic, renal, or thyroid disorders; pediatric patients with obesity; pediatric patients presenting with infectious diseases or who had engaged in excessive physical exercise before sampling; pediatric patients taking drugs that interfere or alter the antioxidant or inflammatory status; pediatric patients taking AEDs prior to the start of the study; and pediatric patients with convulsive crisis leading to difficult handling. The elimination criteria were as follows: pediatric patients whose initial diagnosis of epilepsy was changed to another disease; pediatric patients with insufficient information for the diagnosis of epilepsy; pediatric patients who did not follow the therapeutic regimen during the study and samples with damaged genetic material; and patients who left the study based on their or their relative's decision. For healthy children, the inclusion criteria were the lack of any type of infection or diseases and no family history of epilepsy or other neurological pathology.

For the determination of GR, GPx, SOD, and CAT activities and MDA, H_2_O_2_, 8-OHdG, and 3-NT levels, we obtained 3–5 mL of peripheral venous blood sample at the time of initial diagnosis (drug-free stage) and after VPA monotherapy (samples at 6 and 12 months of AED administration) from the epileptic children. In healthy children, a single blood sample was taken. The samples were collected in EDTA-containing Vacutainer tubes, and plasma samples were obtained by centrifugation at 1500 *×g* for 10 minutes at 4°C. The samples were stored at −80°C until use.

### 2.2. Antioxidant Marker Determination

#### 2.2.1. Glutathione Reductase Activity Assay

The activity of GR was assayed using a Glutathione Reductase Activity Kit (Enzo Life Sciences®, Butler Pike Plymouth Meeting, PA, USA). GR, together with its cofactor NADPH, catalyzes the reduction of oxidized glutathione (GSSG) to glutathione (GSH). In this assay, the oxidation of NADPH to NADP+, during the GR reaction, is spectrophotometrically monitored by the decrease in absorbance (range of 1.7 to 0.2). The activity of GR is expressed in terms of NADPH oxidation. In brief, 50 *μ*L of plasma, blank or GR standard; 100 *μ*L of Master Mix (10X GR buffer, GSSG reagent, and distilled water); and 100 *μ*L of NADP solution were added to initiate the reaction per well. The plate was immediately transferred to an Epoch ELISA reader (BioTek, Winooski, VT, USA), and the absorbance at 340 nm was measured every minute for 10 minutes at room temperature. For the activity calculation, one unit of GR was considered to oxidize 1 *μ*mol of NADPH per minute at 25°C, pH 7.5, and the molar extinction coefficient (*E*) for NADPH was considered 3.732 × 10^−3^ mL/nmol. The data are expressed in U/mL.

#### 2.2.2. Glutathione Peroxidase Activity Assay

The activity of GPx was assayed using a Glutathione Peroxidase Activity Kit (Enzo Life Sciences®, Butler Pike Plymouth Meeting, PA, USA). GPx catalyzes the reduction of H_2_O_2_ to H_2_O using GSH as a coenzyme. GR then reduces the GSSG produced to complete the cycle. The oxidation of NADPH is accompanied by a decrease in absorbance. This rate of decrease is directly proportional to the GPx activity in the sample. In brief, 20 *μ*L of plasma, blank or GPx standard; 20 *μ*L of 10X reaction mix; 140 *μ*L of 1X assay buffer; and 20 *μ*L of cumene hydroperoxide were added to initiate the reaction per well. The absorbance was immediately measured at 340 nm every minute for 10 minutes at room temperature. For activity calculation, one unit of GPx was considered to oxidize 1 nmol of NADPH to NADP+ per minute at 25°C, and the molar extinction coefficient (*E*) for NADPH was considered 3.732 × 10^−3^ mL/nmol. The data are expressed in U/mL.

#### 2.2.3. Superoxide Dismutase Activity Assay

The activity of SOD was assayed using a Superoxide Dismutase Activity Kit. SOD catalyzes the conversion of superoxide radical anion (O_2_·−) to H_2_O_2_ and O_2_. In this colorimetric assay, O_2_·− is generated from the xanthine oxidase reaction. The O_2_·− then converts the WST-1 molecule to formazan, and this colored product is spectrophotometrically detected. In brief, 25 *μ*L of plasma, blank or SOD standard; 150 *μ*L of Master Mix (10X SOD buffer, WST-1 reagent, xanthine oxidase, and distilled water); and 25 *μ*L of 1X xanthine solution were added to initiate the reaction per well. The absorbance was immediately measured at 450 nm every minute for 10 minutes at room temperature. For activity calculation, an inhibition curve for the SOD standard was plotted using %inhibition versus log (units/well SOD standard), and the percentage of inhibition of the rate of change in absorbance was determined at 450 nm in the absence of SOD. The data are expressed in U/mL.

#### 2.2.4. Catalase Activity Assay

The activity of CAT was assayed using a Catalase Activity Kit. CAT catalyzes the conversion of H_2_O_2_ to H_2_O. In this assay, fluorescent resorufin is detected as the reaction product between H_2_O_2_ and a nonfluorescent detection reagent + peroxidase. In brief, 50 *μ*L of plasma, blank or CAT standard, and 50 *μ*L of 40 *μ*M of H_2_O_2_ solution were added to each well, and the plate was incubated for 30 minutes at room temperature. Then, 100 *μ*L of the reaction cocktail (detection reagent, 100X HRP, and 1X reaction buffer) was added to the well, and the plate was incubated for 10 minutes. Fluorescence was measured using excitation and emission wavelengths of 530 and 590 nm, respectively. For activity calculation, a plot of the average net fluorescence units for each standard of CAT standard curve versus CAT activity was constructed. The concentration in each sample was determined by interpolation. The data are expressed in U/mL.

#### 2.2.5. Oxidation Marker Determination

*(1) MDA Level Determination*. The concentration of MDA was measured by interpolating results in a trimethoxypropane standard curve. MDA is a final product of lipid peroxidation that reacts with thiobarbituric acid (TBA) to form thiobarbituric acid reactive species (TBARS). In brief, the reaction mixture consisted of 0.026 M TBA, 0.211 M HCl, 6.66% trichloroacetic acid, and 1 mM deferoxamine mesylate. Each of the different samples (50 *μ*L) was added to each well, and 450 *μ*L of the reaction mixture was added and heated at 100°C for 10 minutes. The mixture was cooled, and the absorbance was measured at 530 nm. The levels of TBARS are expressed as ng of MDA/mL (modified from [25]).

*(2) Hydrogen Peroxide Level Determination*. H_2_O_2_ levels were assayed using a Hydrogen Peroxide Colorimetric Detection Kit. H_2_O_2_ is a reactive oxygen metabolite by-product that serves as a key regulator for a number of oxidation-reduction states and has been linked to a number of degenerative and inflammatory diseases. The kit contains a reagent that contains a dye, xylenol orange, sorbitol, and ammonium iron sulfate, which reacts to produce a purple color proportional to the concentration of H_2_O_2_ in the sample. The exact reaction mechanism is not known. In brief, 50 *μ*L of plasma, blank or H_2_O_2_ standard; 50 *μ*L of sample diluent; and 100 *μ*L of color reagent were added per well, and the plate was incubated for 30 minutes at room temperature. The absorbance was measured at 550 nm. H_2_O_2_ levels are expressed in ng/mL.

*(3) 8-Hydroxy-2-deoxyguanosine Level Determination*. 8-OHdG levels were assayed using a DNA Damage ELISA Kit (Enzo Life Sciences®, Butler Pike Plymouth Meeting, PA, USA). 8-OHdG is a modified nucleoside base that is the most commonly studied and detected by-product of DNA damage linked to many degenerative diseases. The kit uses an anti-8-OHdG monoclonal antibody to bind, in a competitive manner, to 8-OHdG in the sample or standard in the wells of the immunoassay plate. Anti-8-OHdG molecules bound to 8-OHdG in the sample or standard are washed away, while those captured by the immobilized 8-OHdG are detected with a secondary antibody conjugated to HRP. The assay uses tetramethylbenzidine substrate (TMB), and the yellow color is spectrophotometrically detected. The intensity of the color is inversely proportional to the concentration of 8-OHdG. In brief, 50 *μ*L of plasma, blank or 8-OHdG standard, and 50 *μ*L of anti-8-OHdG were added per well and incubated at room temperature for 1 h. The plate was washed 6 times with 1X wash buffer, and 100 *μ*L of HRP-conjugated anti-mouse IgG was added to each well, and the plate was incubated at room temperature for 1 h. Then, the plate was washed as before, and 100 *μ*L of TMB substrate was added per well. The plate was incubated at room temperature for 15 minutes in the dark, and 100 *μ*L of stop solution was added per well. The absorbance was immediately measured at 450 nm. 8-OHdG levels are expressed in ng/mL.

*(4) 3-Nitrotyrosine Level Determination*. 3-NT levels were assayed using the DRG Nitrotyrosine ELISA Kit (DRG Diagnostics, GmbH, Frauenbergstr, Marburg, Germany). 3-NT is the nitrated form of the amino acid tyrosine synthesized from nitric oxide (NO·) and is associated with neurological diseases and cardiovascular diseases based on inflammatory processes. In this kit, a plate coated with polyclonal goat anti-NT antibody is used. Nitrated proteins are bound by the immobilized primary antibody. Then, a peroxidase-conjugated polyclonal goat anti-human protein antibody is added, and a complex primary antibody-nitrated protein-peroxidase conjugate is formed. TMB is used as a peroxidase substrate, and an acidic stop solution is added to terminate the reaction. The intensity of color is directly proportional to the concentration of NT. In brief, the precoated plate was washed 5 times with ELISA wash buffer, and 100 *μ*L of sample, blank or 3-NT standards, was added, and the plate was incubated at room temperature for 1 h. Then, the plate was washed as before, and 100 *μ*L of the conjugate was added per well, and the plate was incubated at room temperature for 1 h. The plate was washed again, and 100 *μ*L of TMB substrate was added per well, and the plate was incubated at room temperature for 20 minutes in the dark. Finally, 100 *μ*L of stop solution was added, and the absorption was immediately determined at 450 nm. 3-NT levels are expressed in ng/mL.

### 2.3. Statistical Analysis

All statistical analyses were performed using the commercially available GraphPad Prism version 6.0 (La Jolla, CA, USA) software and XLSTAT for Excel 2018 (Addinsoft, NY, USA). The data are expressed as the mean ± standard deviation (SD). The Kolmogorov-Smirnov normality test was performed based on the null hypothesis that the distribution is normal. Differences between groups were tested using unpaired *t*-tests and an ANOVA with Bonferroni post hoc analysis, and a correlation analysis was performed using the Pearson test. Statistical significance was assessed at a significance level of *p* < 0.05.

## 3. Results

### 3.1. Patient Characteristics

The characteristics of the patients with epilepsy are presented in Table 1. The mean actual age was 6.4 years (SD = 4.1, range of 2–15 years), and the age at the beginning of VPA treatment was 4.2 years (SD = 4.1, range of 0–12 years). Among the patients, 28.1 and 71.9% were females and males, respectively. In terms of family history of epilepsy, 18.8% of patients had relatives with epilepsy (grandparents, mother, brothers, cousin, or aunt); 1.5% were prematurely born; approximately half of the patients had generalized epilepsy and idiopathic etiology; 12.5% had comorbidities (1 patient had headaches, 1 patient had bronchopulmonary dysplasia, 1 patient had cranial malformation, and 1 patient had anorectal malformation and esophageal atresia); 37.5% of patients had abnormal brain findings on imaging studies (5 patients with atrophy, 2 patients with asymmetry, and 5 patients with other findings); and only 9.4% of patients had partially controlled epilepsy with VPA monotherapy.

### 3.2. Antioxidant Enzyme Activities and Oxidative Marker Levels in the Study Population

Significant decreases in all antioxidant enzyme activities, with the exception of GPx, and increases in all oxidant markers in epileptic subjects in comparison with healthy children were observed. Antioxidant enzyme activities were significantly increased, and all oxidant markers were decreased after of VPA monotherapy, and a differential profile of antioxidant and oxidative markers after 6 and 12 months of VPA monotherapy in comparison with that before therapy was also observed.

In epileptic children, significant decreases in GR, CAT, and SOD activities, diminishing 4.8-, 3.7-, and 3.2-fold, respectively, were also observed, as was a 1.5-fold increase in GPx activity compared with those of healthy children (*p* < 0.0001 for each). After treatment, the epileptic children showed significant increases in GR, SOD, and CAT activities but a significant decrease in GPx activity in comparison with epileptic children before VPA monotherapy. GR activity increased approximately 4-fold, SOD activity increased 4.9- and 4-fold, CAT activity increased 2.4- and 5.6-fold, and GPx decreased approximately 2-fold after 6 and 12 months of VPA treatment, respectively, in comparison with before treatment (*p* < 0.0001 for each). SOD and CAT showed higher activity levels at 6 and 12 months of VPA, respectively (1.2- and 2.4-fold, respectively) in comparison with the levels at 12 and 6 months, respectively (*p* < 0.0001 for each). GPx showed a higher activity at 12 months (1.1-fold) in comparison with the level at 6 months (*p* = 0.0089) (see Table 2). The differences between antioxidant activities were analyzed using one-way ANOVA (*p* < 0.0001, *F* = 245.7 for GR; *p* < 0.0001, *F* = 262.5 for GPx; *p* < 0.0001, *F* = 472.6 for SOD; and *p* < 0.0001, *F* = 1209 for CAT).

In epileptic children, increases of 1.4-, 346-, 8.5-, and 1.6-fold in MDA, H_2_O_2_, 8-OHdG, and 3-NT levels, respectively, compared with those of healthy children were observed (*p* < 0.0001 for each). After treatment, the epileptic children showed significant decreases in all oxidant marker levels in comparison with epileptic children before VPA monotherapy. MDA levels decreased 1.4- and 1.8-fold, H_2_O_2_ levels decreased 3.5- and 2.7-fold, 8-OHdG levels decreased 2- and 1.2-fold, and 3-NT levels decreased approximately 1.6-fold after 6 and 12 months of VPA treatment, respectively, in comparison with before treatment (*p* < 0.0001 for each). MDA levels showed lower levels (1.3-fold) and H_2_O_2_ and 8-OHdG showed higher levels (1.3- and 1.6-fold, respectively) at 12 months of VPA treatment in comparison with the 6-month levels (*p* < 0.0001 for each) (see Table 2). The differences between oxidative marker levels were analyzed using one-way ANOVA (*p* < 0.0001, *F* = 73.19 for MDA; *p* < 0.0001, *F* = 18333 for H_2_O_2_; *p* < 0.0001, *F* = 392 for 8-OHdG; and *p* < 0.0001, *F* = 226 for 3-NT).

### 3.3. Relationships between Antioxidant and Oxidative Markers with Some Clinicopathologic Characteristics

#### 3.3.1. Comparative Analysis

A comparative analysis between antioxidant enzyme activities and oxidative marker levels and the presence or absence of brain findings on imaging studies in untreated epileptic patients and between the effectiveness of crisis control by VPA during the first 6 months of monotherapy was performed. In relation to antioxidant enzyme activities, a significant decrease of 1.1-fold in SOD activity in patients with abnormal brain findings on imaging studies was observed in comparison with that in patients without these findings (*p* = 0.0067).

In relation to oxidative marker levels, significant increases of approximately 1-fold in H_2_O_2_ levels (*p* = 0.0002) and 8-OHdG levels (*p* = 0.009) in patients with brain findings on imaging studies were observed in comparison with those in patients without these findings. With respect to the crisis control by VPA in relation to antioxidant enzyme activities, significant decreases of approximately 1-fold in GR, GPx, and CAT activities in patients with absolute control were observed in comparison with those in patients with partial control (*p* < 0.0001 for GR and GPx and *p* = 0.0001 for CAT). In relation to oxidative marker levels, a significant decrease of 1.2-fold in MDA levels and significant increases of 1.3-fold in 8-OHdG levels in patients with absolute control were observed compared with those in patients with partial control (*p* < 0.0001) (see Table 3).

Additionally, a comparative analysis between antioxidant enzyme activities and oxidative marker levels and the type and etiology of epilepsy in untreated epileptic patients was performed. With respect to the type of epilepsy, significant decreases of 1.2-fold in SOD activity (*p* < 0.0001) and 1.1-fold in 8-OHdG levels (*p* = 0.0014) and significant increases of 1.1-fold in MDA levels (*p* = 0.0017) and 3-NT levels (*p* = 0.0226) in focal epilepsy were observed in comparison with those in generalized epilepsy. With respect to the etiology of epilepsy, significant decreases of 1.1-fold in CAT activity (*p* < 0.0001) and of 1.2-fold in 3-NT levels (*p* < 0.0001) in symptomatic epilepsy were observed in comparison with those in idiopathic epilepsy (see Table 4).

#### 3.3.2. Correlation Analysis

The correlations of antioxidant enzyme activities and oxidative marker levels with the age of children at the beginning treatment, number of seizures before VPA monotherapy, number of seizures after VPA monotherapy, weight-based divided dose of VPA, and serum levels of VPA were determined; only the statistically significant results are shown.

GPx activity before VPA monotherapy was positively correlated with age at the beginning of treatment (*p* = 0.0262, *r* = 0.3259). However, SOD activity after 12 months of VPA monotherapy was positively correlated with age at the beginning of treatment (*p* = 0.0331, *r* = 0.6302). H_2_O_2_ levels after 12 months of VPA monotherapy were positively and strongly correlated with age at the beginning of treatment (*p* = 0.0428, *r* = 0.9162) and with the number of seizures before VPA monotherapy (*p* = 0.0328, *r* = 0.9355). Additionally, 8-OHdG levels after 6 months of VPA monotherapy were positively and strongly correlated with age at the beginning of treatment (*p* = 0.0249, *r* = 0.9509).

## 4. Discussion

The major findings of the present work were as follows: (1) We observed significant decreases in all antioxidant enzyme activities, with the exception of GPx, and increases in all oxidant markers in epileptic children compared with those in healthy children. GR, SOD, and CAT activities were significantly increased, and all oxidative marker levels were decreased after VPA monotherapy in comparison with the corresponding activities and levels before treatment. We also observed significant differential profiles between 6 and 12 months of VPA monotherapy in GPx, SOD, and CAT activities and in MDA, H_2_O_2_, and 8-OHdG levels. (2) We observed significant differences in some antioxidant enzyme activities and some oxidative marker levels depending on the presence or absence of brain findings on imaging studies and type and etiology of epilepsy in untreated epileptic patients and between the effectiveness of crisis control by VPA during the first 6 months of monotherapy in epileptic patients. (3) We observed positive and significant correlations of GPx and SOD activities and H_2_O_2_ and 8-OHdG levels with the age of children at the beginning of treatment. H_2_O_2_ levels were also positively correlated with the number of seizures before VPA monotherapy. All results indicate that VPA exerts an antioxidative effect and that these findings may facilitate a new understanding of the neuroprotective mechanism of VPA in epileptic children.

Oxidative stress (OS) is defined as a biochemical state in which reactive oxygen species (ROS) are generated. This state is involved in many chronic diseases, such as diabetes, atherosclerosis, inflammatory cancer, and neurological diseases [26]. OS was first associated with neuronal hyperexcitation by Dalton and his group in a rat model of epilepsy [27]. To date, many clinical studies have demonstrated the relation between ROS generation and the presence of convulsions contributing to disease progression [28–30]. Moreover, clinical studies have shown the neuroprotective role of some AEDs (phenytoin, phenobarbital, carbamazepine, oxcarbazepine, levetiracetam, and VPA) [31]; however, the antioxidant effect of these drugs is still controversial. In many clinical studies, VPA has been shown to have an antioxidative effect, but in others, an oxidative effect: VPA increases lipoperoxidation, 8-OHdG, nitrite/nitrate, GSH, and selenium (Se) levels and SOD, GPx, GR, and xanthine oxidase activities and diminishes Se levels, total antioxidant capacity, and SOD, GPx, and GR activities in plasma, serum, urine, leukocytes, or erythrocytes [15, 16, 19, 21–24, 30, 32–43]. These controversial results are evidence of the relevance of defining the study population, directing the studies to the type of crisis and the sampling times, together with the adequate and defined description of the therapeutic approach of the said populations.

This is the first report where H_2_O_2_ and 3-NT levels were measured in epileptic children and where antioxidant (GR, GPx, SOD, and CAT) and oxidant markers (MDA, H2O2, 8-OHdG, and 3-NT) were measured in a longitudinal study, showing a differential redox profile with the presence and absence of brain findings on imaging studies, between the type and etiology of epilepsy in untreated epileptic children and between the effectiveness of crisis control by VPA during the first 6 months of monotherapy. The brain seizure activity is characterized by intense activation of mitochondrial oxidative phosphorylation accompanied by an increase in ROS formation [44], but it is still unclear which ROS are attributed to the convulsive phenomenon and if the AEDs can contribute to decreasing the OS phenomenon as a mechanism of action. In a past work, we reported that topiramate has an *in vitro* antioxidant effect [45]. In this work, we showed, for the first time, that antioxidant/oxidant status is dependent on the clinical status of the epileptic patient, principally with the age and the presence of structural brain alterations, that the oxidation of the principal biomolecules (DNA, lipids, and proteins) is present in the epileptic condition, and that VPA acts as an antioxidant in epileptic children; however, this mechanism is modulated differentially throughout the 12 months of monotherapy treatment. Moreover, it has been demonstrated that H_2_O_2_ production is related to the presence of seizures in patients with focal and generalized epilepsy with principally idiopathic etiology. VPA has shown beneficial effects in mitochondria, exhibiting a neuroprotective effect against oxidative damage-induced cell death but only using experimental models of epilepsy [46]. As mitochondria are the principal source of ROS, among them H_2_O_2_ [47], our study suggests that VPA could be more efficacious in juvenile epileptic patients with overexpression of genes related to mitochondrial oxidative phosphorylation.

The results reported in this work are consistent with some reports in the literature where decreases in the activity levels of some antioxidant enzymes in epileptic patients (GR, SOD, or CAT) in comparison with healthy subjects were shown and where this activity is restored after of treatment with AED (phenobarbital or VPA) [48, 49]. The results obtained in this study together with the results of these authors suggest that the increase in H_2_O_2_ levels increased GPx activity and lipoperoxidation levels in epileptic patients before AED treatment. This increase in H_2_O_2_ levels in epilepsy also could explain the specific increase in GPx activity and the decrease of SOD activity [50].

The increase in lipoperoxidation levels in epilepsy has been shown in many clinical studies [17, 30, 32, 35, 48, 51, 52], suggesting that oxidative lipid damage from seizure activity plays an important role in seizure-induced death of vulnerable neurons. The results showed that VPA treatment diminished MDA levels, as is reported [21]. In this sense, the protective effect of VPA can explain the overexpression of receptors involved in the metabolism of fatty acids [53]. Of note, in this study, a significant increase in 8-OHdG levels in epileptic patients was observed, although VPA significantly decreased the levels of this oxidative marker to almost half after 6 months of treatment. This observation is consistent with clinical studies in which 8-OHdG levels are increased in patients with seizures [37, 54, 55]. However, until now, only one study determined the effect of monotherapy with VPA on 8-OHdG levels and showed that after 60 days of VPA monotherapy, 8-OHdG levels were increased in the sera of children with therapeutic VPA levels compared with those of healthy subjects. The authors concluded that VPA induces DNA damage, although where 8-OHdG is produced in patients treated with AED remains unknown [33]. The results of our work indicate that VPA could protect against DNA oxidative damage when it is administered for at least 6 months. Moreover, the significant decrease of 3-NT levels with VPA monotherapy shows that the antiepileptic effect of VPA may be mediated through the capacity of VPA to release NO· and a possible decrease in thiol oxidation diminishing neuronal damage, as suggested in some studies conducted in patients [41, 54].

The results obtained in this work also showed relationships between antioxidant/oxidant markers and some clinicopathological factors. Moderate increases in SOD activity and 8-OHdG levels in generalized epilepsy and CAT activity and 3-NT levels in idiopathic epilepsy were observed, indicating that the neurovascular unit and blood-brain barrier dysfunction increases OS and that the neuroinflammation present in epilepsy differs with brain regions, as has been observed in some clinical studies [56–59]. The presence of cerebral structural alterations modifies the rate of antioxidant/oxidant responses in the brain of epileptic children, increasing the susceptibility to oxidative damage with increasing H_2_O_2_ levels. The evidence also suggests that the presence of lipoperoxidation in epilepsy is related to the effect of the AED response diminishing the efficacy of VPA during the first 6 months of monotherapy.

Significant and positive correlations of some enzyme antioxidant activities (GPx and SOD) and OS marker levels (H_2_O_2_ and 8-OHdG) with age at the beginning of VPA treatment were also shown. H_2_O_2_ levels were also highly correlated with the number of seizures before VPA monotherapy. Evidence in the literature has shown that the degree of brain damage is highly age-dependent, that oxidative DNA damage increases in the mature brain, and that SOD activity is altered in immature brain during seizure activity [60–64]. These observations and the results obtained in this work support the idea that a young brain has lower superoxide production (and possibly other ROS) than a more mature brain and that H_2_O_2_ plays a principal role in increasing seizures in children with epilepsy. In a recent work, microarray technology was used to show that VPA is a neuroprotector since it has antioxidant properties during the first 6 months of treatment, modulating oxidative stress, glutathione metabolism, selenium metabolism, and selenoprotein pathways, including the gene expression of some respiratory chain proteins [9], suggesting that mitochondria may be a direct or indirect therapeutic target of VPA when used during the management of pediatric epilepsy.

With all the previous findings, we must consider that the effects that occur during oxidant stress are associated with the increase in the glutamatergic response that favors the neuronal hyperexcitability that underlies epileptic seizures. Thus, VPA, by positively modulating the antioxidant enzymes, modulates hyperexcitability either directly or secondarily to the modulation of the glutamatergic response, favoring the control of epileptic seizures. It should be mentioned that VPA presents a large gamma of mechanisms already reported, but this study is the first evidence that demonstrates its effect on biochemical markers of oxidative stress, longitudinally, in the pediatric population, contributing to the biochemical characterization of this population and allowing us to provide information that can be used to understand the complexity of the effects of this drug in other populations diagnosed with epilepsy.

## 5. Conclusions

VPA shows significant antioxidative effects by decreasing MDA, H_2_O_2_, 8-OHdG, and 3-NT oxidative marker levels and increasing GR, SOD, and CAT activities. This property might lead to a decrease in seizure activity resulting in AED efficacy and a simultaneous neuroprotective effect by ROS modulation, possibly in a time-dependent manner. Moreover, our results suggest that the presence of cerebral structural alterations, the effectiveness of crisis control by VPA, and age are related to the redox status in children with epilepsy, although the type and etiology of epilepsy can also influence the effect of VPA.

## Figures and Tables

**Table 1:** Clinicopathological characteristics of the epileptic patients.

Characteristic	Total (*n* = 32)
Actual age (years, mean ± SD)	6.4 ± 4.1
Age at the beginning of treatment (years, mean ± SD)	4.2 ± 4.1
Sex (%)	Female (9): 28.1
	Male (23): 71.9
BMI (kg/m^2^, mean ± SD)	16.9 ± 2.7
Family history of epilepsy (%)	Yes (6): 18.8
	No (21): 65.6
	Unknown (5): 15.6
Type of epilepsy (%)	Focal (15): 46.9
	Generalized (17): 53.1
Etiology of epilepsy (%)	Symptomatic (6): 18.7
	Idiopathic (26): 81.3
Comorbidities (%)	Neurological (1): 3.1
	Pulmonary (1): 3.1
	Malformations (2): 6.3
Brain findings on imaging studies using AT and MRI (%)	Yes (12): 37.5
	No (20): 62.5
Crisis control by VPA (%)	Absolute (29): 90.6
	Partial (3): 9.4
Serum levels of VPA (*μ*g/mL, mean ± SD)	28.9 ± 16.9
Weight-based divided dose of VPA (mg/kg, mean ± SD)	24.04 ± 9.34

BMI: body mass index; AT: computerized axial tomography; MRI: magnetic resonance imaging.

**Table 2:** Antioxidant enzyme activities and oxidative marker levels in patients vs. healthy controls.

Study groups				
Enzyme	Control	Before treatment	6 months of VPA treatment	12 months of VPA treatment
Antioxidant enzyme activities (U/mL, mean ± SD)				
GR	154.9 ± 32.4	32.1 ± 6.8^a^	137.3 ± 16^b,d^	131.5 ± 21^b,d^
GPx	81.2 ± 5.7	122.9 ± 12.9^a^	63.8 ± 8.6^a,d^	71.3 ± 6.2^b,d,e^
SOD	37.7 ± 5.2	10.3 ± 1.9^a^	50.5 ± 5.6^a,d^	42.1 ± 4.8^b,d,f^
CAT	99 ± 13.5	31 ± 3.3^a^	73.1 ± 8.3^a,d^	175.4 ± 13.1^a,d,f^
Oxidative stress marker levels (ng/mL, mean ± SD)				
MDA	5973.2 ± 866.8	8219.8 ± 1423.2^a^	5881 ± 895.3^d^	4658.5 ± 389.6^c,d,f^
H_2_O_2_	113.4 ± 19.7	39283.3 ± 371^a^	11,196 ± 670.9^a,d^	14559.5 ± 877.4^a,d,f^
8-OHdG	7.7 ± 0.8	65.7 ± 8.9^a^	33.4 ± 5.8^a,d^	55.2 ± 4^a,d,f^
3-NT	16.5 ± 1.1	27.1 ± 3.3^a^	16.6 ± 0.9^d^	16.5 ± 0.9^d^

^a^ *p* < 0.0001, ^b^*p* < 0.02, and ^c^*p* = 0.0002 vs. control; ^d^*p* < 0.0001 vs. before treatment; ^e^*p* = 0.0089 and ^f^*p* < 0.0001 vs. 6 months of VPA treatment. GR and GPx activity assays were performed in duplicate, and CAT and SOD activity assays were performed in triplicate. H_2_O_2_, 8-OHdG, and 3-NT levels were determined in duplicate. MDA levels were determined in triplicate.

**Table 3:** Comparative analysis between antioxidant enzyme activities and oxidative marker levels and the presence or absence of brain findings on imaging studies in untreated epileptic patients and between the effectiveness of crisis control by VPA during the first 6 months of monotherapy in epileptic patients.

Study groups				
Enzyme	Brain findings on imaging studies		Crisis control by VPA (6 months)	
	No	Yes	Absolute	Partial
Antioxidant enzyme activities (U/mL, mean ± SD)				
GR	33.6 ± 7.9	32.1 ± 8.3	132.4 ± 14.1^d^	157 ± 18
GPx	125.3 ± 14.2	122.7 ± 13	62.6 ± 8.3^d^	73.5 ± 5.4
SOD	10.6 ± 1.7	9.1 ± 2.5^a^	51 ± 5	48.7 ± 10.8
CAT	30.9 ± 3.6	30.6 ± 2.5	69.1 ± 8.3^e^	78 ± 9.1
Oxidative stress marker levels (ng/mL, mean ± SD)				
MDA	7990 ± 1509	8520 ± 1300	5700 ± 900^d^	6694 ± 830
H_2_O_2_	39,110 ± 389.5	39456.6 ± 308^b^	11,203 ± 749.9	11,161 ± 810
8-OHdG	63.1 ± 10	69 ± 7.3^c^	37.5 ± 7.2^d^	29.4 ± 5.8
3-NT	26.1 ± 4.2	25.6 ± 2.8	16.6 ± 0.6	16.9 ± 1.5

^a^ *p* = 0.0067, ^b^*p* = 0.0002, and ^c^*p* = 0.009 vs. patients with brain findings on imaging studies; ^d^*p* < 0.0001 and ^e^*p* = 0.0001 vs. partial crisis control by VPA.

**Table 4:** Comparative analysis between antioxidant enzyme activities and oxidative marker levels and the type and the etiology of epilepsy in untreated epileptic patients.

Study groups				
Enzyme	Type		Etiology	
	Focal	Generalized	Symptomatic	Idiopathic
Antioxidant enzyme activities (U/mL, mean ± SD)				
GR	33 ± 6.1	31.6 ± 7.6	31.6 ± 7.7	32.2 ± 6.8
GPx	121.3 ± 14	124 ± 13	121.4 ± 12	123.1 ± 13.4
SOD	9.4 ± 2^a^	11.3 ± 1.4	10.7 ± 0.9	10.2 ± 2.1
CAT	31.9 ± 2.5	30.4 ± 3.5	28.4 ± 2.5^e^	31.4 ± 3.2
Oxidative stress marker levels (ng/mL, mean ± SD)				
MDA	8832.5 ± 1141.7^c^	7751.2 ± 1468.1	7755.2 ± 876.2	8291.2 ± 1489.1
H_2_O_2_	39252.2 ± 428.5	39,330 ± 343.4	39,230 ± 510	39,289 ± 400.5
8-OHdG	60.3 ± 12^b^	68.4 ± 6.6	66.9 ± 4.6	65.4 ± 10.1
3-NT	28.8 ± 5^d^	26.1 ± 4.2	23.2 ± 1^e^	28.6 ± 0.2

^a^ *p* < 0.0001, ^b^*p* = 0.0014, ^c^*p* = 0.0017, and ^d^*p* = 0.0226 vs. generalized epilepsy; ^e^*p* < 0.0001 vs. idiopathic epilepsy.

## Data Availability

The data used to support the findings of this study are available from the corresponding author upon request.
